# Ilheus Virus (ILHV) Resistance in *Culex quinquefasciatus* from the Northern Region of Brazil

**DOI:** 10.3390/life14040427

**Published:** 2024-03-22

**Authors:** Lúcia Aline Moura Reis, Ana Beatriz Oliveira Pampolha, Daniel Damous Dias, Maissa Maia Santos, Jamilla Augusta de Sousa Pantoja, Pedro Arthur da Silva Araújo, Fábio Silva da Silva, Bruna Lais Sena do Nascimento, Valéria Lima Carvalho, Eliana Vieira Pinto da Silva, Joaquim Pinto Nunes Neto

**Affiliations:** 1 Graduate Program in Parasitary Biology in the Amazon Region, Center of Biological and Health Sciences, State University of Pará, Belém 66095-663, Brazil; 2 Institute of Biological Sciences, Faculty of Biological Sciences, Federal University of Pará (UFPA), Belém 66075-110, Brazil; 3 Department of Arbovirology and Hemorrhagic Fevers, Evandro Chagas Institute—IEC/MS/SVSA, Ananindeua 67030-000, Brazilelianapinto@iec.gov.br (E.V.P.d.S.); 4 Graduate Program in Biology of Infectious and Parasitary Agents, Institute of Biological Sciences, Federal University of Pará (UFPA), Belém 66077-830, Brazil

**Keywords:** arbovirus infections, flavivirus, insect vectors, *Culex*, virus replication, Ilheus virus

## Abstract

Background: *Orthoflavivirus ilheusense* (ILHV) is a member of the Flaviviridae family. It was first isolated in 1944 from pools of *Aedes serratus* and *Psorophora ferox* mosquitoes; however, it has also been detected in species of the genus *Culex*, such as *Cx*. *portesi* and *Cx*. *coronator*. The objective of this study was to examine the vector competence of *Cx*. *quinquefasciatus* mosquitoes to ILHV infection and the subsequent transmission of the virus through their saliva during feeding on blood. Methods: F1 generation females of *Cx*. *quinquefasciatus* (Ananindeua/PA) were orally infected with goose blood infected with strain BeH7445, and body, head and saliva samples were analyzed at 7, 14, and 21 dpi using the techniques of virus isolation in cells and indirect immunofluorescence. Results: The presence of ILHV was not detected in the body and head samples of *Cx*. *quinquefasciatus* females at any of the three dpi’s analyzed, indicating that the lineage of mosquitoes analyzed was resistant to ILHV. Conclusions: According to the results obtained in this study, the species *Cx*. *quinquefasciatus* proved resistant to ILHV, regardless of the virus titers to which it was exposed, which suggests the possibility that this species does not act as a vector in the ILHV transmission cycle.

## 1. Introduction

*Orthoflavivirus ilheusense* (ILHV) is an arbovirus that belongs to the *Flaviviridae* family and the Orthoflavivirus genus [1]. It was first identified in 1944 from pools of *Aedes serratus* and *Psorophora ferox* mosquitoes collected at the Pirataquissé farm near Ilhéus city (BA) and was first named “Ilhéus encephalitis virus” [2].

ILHV is currently classified with other flaviviruses, including *Orthoflavivirus bagazaense* (BAGV), *Orthoflavivirus israelense* (ITV), *Orthoflavivirus tembusu* (TMUV), and *Orthoflavivirus zikaense* (ZIKV), as belonging to the *Orthoflavivirus ntayaense* (Ntaya virus—NTAV) antigenic group [1,3].

In view of the first isolation, Laemmert Jr. and Hughes [2] attempted to study the transmission of ILHV by mosquitoes given its isolation from arthropod samples. For this purpose, colonies of different species, *Ae*. *aegypti*, *Ae*. *serratus,* and *Ps. ferox*, were tested for transmissibility. The results showed that the species were capable of transmitting ILHV to naive mice.

The transmission cycle of ILHV is comparable to that of other enzootic arboviruses, with Ps. ferox mosquitoes being the principal vector. However, secondary vectors are also considered, including mosquitoes of the genera *Aedes*, *Culex*, *Haemagogus*, *Trichoprosopon*, and *Ochlerotatus* [4,5,6]. Birds act as amplifying hosts and humans act as incidental hosts, also known as terminal hosts, because they do not produce viremia high enough to allow the virus to be transmitted to other arthropods [7,8,9].

Since its first isolation, ILHV has been detected in several species of wild birds, mainly *Columbina talpacoti* [10,11]. It has also been identified in nonhuman primates (NHPs), such as marmosets of genus *Callithrix* [11], and in capuchin monkeys of genus *Sapajus*, such as *Sapajus libidinosus* and *Sapajus flavius* [12].

Regarding the detection of ILHV in arthropods, Woodall [13] identified the arbovirus in different species of mosquitoes of the genus *Aedes* and *Psorophora*, such as *Ae*. *fulvus*, *Ae*. *leucocelaenus*, *Ae*. *scapularis*, *Ae*. *serratus*, *Ps*. *albipes*, and *Ps*. *ferox*. Pauvolid-Corrêa et al. [14] also identified ILHV in *Ae*. *scapularis* samples collected in Nhecolândia Pantanal (MS), a region belonging to the Pantanal Complex, and Araújo et al. [4] identified the virus in *Culex* (*Melanoconion*) *portesi* samples collected in the Caxiuanã National Forest in the municipality of Melgaço (PA).

With respect to the detection of ILHV in mosquitoes of the *Culex* genus, Cunha et al. [5] reported the isolation of the virus in pools of *Culex* spp. pools collected in Santo Antônio do Aracanguá (SP) city in 1994, and Vieira et al. [6] also identified ILHV by RT-PCR in two pools of *Culex* females, one with *Cx*. *coronator* and the other with *Cx*. (*Mel*.) sp. collected in the cities of Sinop (MT) and Ipiranga do Norte (MT), respectively.

The *Culex quinquefasciatus* species, commonly known as the “house mosquito”, has emerged as an important arbovirus vector in urban areas, particularly where anthropogenic interventions have significantly altered the peridomiciliary environment. The species has adapted extensively to the urban environment and can be readily located in both human and animal habitats where it locates breeding sites, as the female deposits her eggs in small pools of stagnant water containing high levels of organic matter [15,16,17].

The *Cx. quinquefasciatus* is considered to be highly anthropophilic; i.e., it has a preference for human blood, so it actively seeks out humans in their homes to carry out its hematophagy. However, it also exhibits ornithophilic behavior, as domestic birds are the second most attacked host after humans by females of the species. In addition, females that feed on human and avian blood have higher egg production compared to those that feed on blood from other sources [15].

Research conducted in the municipalities of Guadalupe and Escobedo, located in northeastern Mexico, aimed to investigate the host selection patterns of *Cx*. *quinquefasciatus*. Mosquitoes were captured both indoors and outdoors. The majority of *Cx*. *quinquefasciatus* females obtained their blood meals from poultry and humans, comprising around 70% of cases. Human blood was detected in a significant proportion of adult female mosquitoes living indoors, illustrating the propensity of these mosquitoes to feed directly on human hosts. Nevertheless, a considerable proportion of mosquitoes also fed on birds, demonstrating the adaptability of this mosquito in choosing its hosts. This host preference duality is an essential element in the ecology of *Cx*. *quinquefasciatus*, as it affects both its population dynamics and its interaction with mosquito-borne pathogens [18].

Blood contains various essential nutrients, including plasma, red blood cells, leukocytes, platelets, hemoglobin, and immunoglobulins, which are vital for mosquitoes. However, blood also serves as a rich source of infection for arthropods since it exposes them to numerous pathogens, including bacteria, fungi, and viruses. Therefore, these animals have evolved several anatomical and physiological barriers to prevent infection [7,19,20].

Vector competence can be defined as the ability of a vector to become infected with a pathogen, indicating susceptibility to infection, to maintain its multiplication in tissues, corresponding to the so-called “extrinsic incubation period” (EIP), and to transmit it to a susceptible host due to high viremia in saliva at the time of the blood meal [21,22,23].

The process of pathogen transmission, known as EIP, involves the ingestion of contaminated blood and subsequent transfer to a new host. Precise coordination of these steps is critical for successful transmission. This requires viral replication within midgut cells, migration through the hemolymph to secondary organs, and ultimately reaching the salivary gland and saliva [22].

Thus, seeking to better understand the affinity of some viruses for certain species of vectors, phylogenetic analysis suggests that flaviviruses are classified based on different factors, including the transmission vector. The evolution of genetic material has permitted these viruses to acquire unique characteristics like cell and tissue tropism [24]. A phylogenetic analysis conducted by Rathore and St. John [24] revealed that the Japanese encephalitis virus complex, the Ntaya virus complex, the Aroa virus complex, and the Kokobera virus complex form a closely related group based on their mode of transmission, which is through mosquitoes of the genus *Culex*, including *Cx. annulirrostris*, *Cx. tritaeniorhynchus*, *Cx. nebulosus*, *Cx. pipiens*, and *Cx. neavei*. The vast majority of these viruses are encephalitogenic.

Although mosquitoes of the genus *Culex* have been associated with the transmission of ILHV, there are no studies in the literature that evaluate the vector competence of any species within this genus for ILHV transmission, and based on the detection records of ILHV in samples of mosquitoes belonging to the *Culex* genus and the analyses presented by Rathore and St. John [24], wherein ILHV was phylogenetically grouped with viruses that this genus transmits, the objective of this study was to examine the vector competence of *Cx. quinquefasciatus* mosquitoes to ILHV infection and the subsequent transmission of the virus through their saliva during feeding on blood.

## 2. Materials and Methods

### 2.1. Ethical Approval

This research was approved by the Ethics Committee for the Use of Animals of the Evandro Chagas Institute (CEUA/IEC) under Certificate No. 03/2022 since blood from Anseriformes was used to carry out the oral infections of the mosquitoes.

### 2.2. Mosquito Colony

Colonies of *Cx*. *quinquefasciatus* (Ananindeua/PA) were provided by the SAARB Medical Entomology Laboratory and maintained at 28 °C ± 1 °C, 80% ± 10% humidity, and 12 h/12 h light/dark cycles [25].

The study used the F1 generation to obtain results closer to those obtained in the external environment. The eggs were placed in basins filled with distilled water. After hatching, larvae (L1 to L4) were fed crushed fish food. Upon pupal emergence, they were transferred to a container of distilled water and placed in entomological cages of 30 cm^3^. As for the adult forms, they were fed a daily with a 10% sugar solution “ad libitum”, diluted in a ratio of 10% pure honey to 90% distilled water [26].

### 2.3. Virus Strain

The BeH 7445 strain used in this study was obtained from the blood sample of an adult male resident in the Japanese colony of Guamá (Belém—Pará) in 1957. It was isolated from the brain of an adult albino Swiss mouse (*Mus musculus*) after 21 passages [27].

### 2.4. Virus Stock

The virus stock was prepared using a culture of VERO (*Chlorocebus aethiops*) cells (ATCC CCL-81) by inoculating 150 µL of virus into a 175 cm^2^ flask containing a monolayer of VERO cells. This flask was then incubated in an incubator (BOD 390 Liter Refrigerated Incubator, SL-117/390, Solab Científica, Piracicaba, São Paulo, Brazil) at 37 °C and 5% CO_2_ for one hour for adsorption, with the flask gently shaken every 15 min to increase virus adsorption. After adsorption, 25 mL of maintenance culture medium 199 (Gibco, Grand Island, NY, USA) supplemented with 2% fetal bovine serum (FBS) (Gibco, Grand Island, NY, USA), 100 UI/mL penicillin, and 100 µg/mL streptomycin was added. The cells were then re-incubated in an incubator and observed under an inverted microscope (ZEISS Stemi 508 Greenough stereomicroscope, Oberkochen, Germany) for seven days to determine the occurrence of cytopathic effect (CPE). Upon detection of CPE in 90% of the monolayer, 2.5 mL FBS (10% of the total maintenance volume) was added and 2 mL aliquots were taken and stored in a freezer at −70 °C until use [28].

### 2.5. Viral Titration

The virus stock, as well as samples of infected blood given to the mosquitoes in the oral infection procedures, were subjected to the viral titration test.

Viral titration was performed according to the protocols of Dulbecco and Vogt [29] and Tesh [28], by serial dilution of samples 10-fold from 10^−1^ to 10^−6^ in a 96-well plate (Kasvi, São José dos Pinhais, Paraná, Brazil), and 225 μL of maintenance culture medium 199 (GIBCO) was added to the wells of the plate and 25 μL of the samples were added to the wells marked “−1”. This mixture was homogenized and 125 μL was transferred from the “−1” wells to the wells marked “−2”, repeating this procedure until the last dilution of “−6”

After dilution, in a 24-well plate (Kasvi, São José dos Pinhais, Paraná, Brazil) containing a monolayer of VERO cells, 100 μL of each dilution was inoculated into each well identified from −1 to −6, with each row of wells corresponding to one sample. The plate was incubated in an oven (BOD 390 Liter Refrigerated Incubator, SL-117/390, Solab Científica, Piracicaba, São Paulo, Brazil) at 37 °C and 5% CO_2_ for 1 h for adsorption, with gentle shaking every 15 min. After adsorption, 1.5 mL of carboxymethylcellulose (CMC, 3% in medium 199) supplemented with 5% FBS, penicillin (100 IU/mL), and streptomycin (100 μg/mL) was added to each well of the plate, and the incubation was repeated for four days in an incubator. After this period, the cells were fixed with 10% formaldehyde by adding 1.5 mL per well and then incubated at room temperature for 24 h, after which the formaldehyde was removed from the plate, washed with running water, and 1.5 mL of 0.1% crystal violet dye (Newprov) was added per well and the incubation was repeated for another 24 h at room temperature.

Viral titers were calculated by multiplying the number of plaque-forming units (PFU) recorded by the 10% dilution factor and by the dilution rate at which the plaques were tabulated. The final result was expressed in plaque-forming units per milliliter (PFU/mL).

Viral titer = number of PFU × dilution factor × dilution,(1)

The titer of the viral stock was 3.1 × 10^7^ PFU/mL.

### 2.6. Oral Infections

Prior to infective blood feeding, five- to seven-day-old female mosquitoes were isolated and deprived of sugar for 24 h. Oral infection was conducted using a Borel bottle equipped with an artificial glass feeder whose blood supply area was wrapped in a bovine liver peritoneal membrane purchased from a local slaughterhouse [25]. The artificial feeder contained 2 mL of defibrinated goose blood supplied by the IEC Bioterium and 2 mL of virus stock. A 500 µL mixture was collected for virus titer analysis. The infected blood sample was heated to 37 °C in a water bath (Digital Bath NI 1255-25L, Nova Instruments, Piracicaba, São Paulo, Brazil) [30,31]. Female mosquitoes were exposed to the infectious blood meal for 60 min, then separated and transferred to entomological cages [32].

### 2.7. Mosquito Segmentation

At 7, 14, and 21 days post-infection (dpi), head and body segmentation (thorax and abdomen) and saliva collection were performed. For head and body segmentation, immobilized infected females were placed on a sterile microscope slide (Kolplast, São Paulo, Brazil) with the abdomen facing up after being paralyzed in an ice bath, and wings and legs were extracted using entomological forceps [15]. To extract saliva, the female’s proboscis was inserted into the tip of a 10 μL polypropylene micropipette (Axygen, São Paulo, Brazil) containing 5 μL FBS (Gibco, Grand Island, NY, USA). The sample was then allowed to stand for 30 min to allow salivation to occur. After this period, the medium containing the saliva was transferred to Epperdorf tubes containing 45 μL Leibowitz L-15 maintenance medium (Gibco, Grand Island, NY, USA) and was immediately stored in a freezer at −70 °C [33,34].

The head and body were transferred to Eppendorf tubes, and 1000 μL Dulbecco’s phosphate-buffered saline (DPBS) (Life Technologies, Carlsbad, CA, USA) containing 2% penicillin and streptomycin, 1% fungizone, and 5% SBF and a 3 mm stainless steel bead were added to the Eppendorf tubes for maceration in a TissueLyser II (Qiagen, Hilden, Germany) at a speed of 25 rotations (Hz) for 1 min. The samples were subsequently preserved at −70 °C until inoculation [15].

### 2.8. Virus Isolation

The samples were centrifuged (Mikro 220R, Hettich, Föhrenstr, Tuttlingen, Germany) at 10,000 revolutions per minute (rpm) for 10 min at 4 °C. A monolayer of C6/36 cells (*Aedes albopictus*) (ATCC: CRL-1660) was then inoculated with 100 μL of the macerate supernatant. The cells were incubated in an incubator at 28 °C and 5% CO_2_ for one hour for adsorption and were gently homogenized every 15 min to promote viral adsorption. Then, 5 mL of Leibowitz L-15 maintenance medium (Gibco, Grand Island, NY, USA) supplemented with tryptose phosphate, non-essential amino acids, penicillin, streptomycin, and 2% FBS was added to the vials and stored in an incubator (Thermo Scientific Napco 8000 CO_2_ Water Jacketed Incubator, Waltham, MA, USA) with 5% CO_2_ at 28 °C (±2 °C) and examined daily under an inverted light microscope (Olympus CK2 Phase Contrast Microscope, Shibuya-ku, Tokyo, Japan) for seven days to check for the occurrence of CPE [35].

### 2.9. Indirect Immunofluorescence Assay

Twenty-five μL of the supernatant of the inoculated cells was added to each well of the slide, the cells were allowed to sediment for 10 min, the excess sample was removed, and the slide was dried in a biological safety cabinet (BSC). The slides were then fixed with propane acetone (PA) for 10 min at −20 °C, and then 25 μL of ILHV-specific polyclonal primary antibody was added, which was obtained from hyperimmune ascites fluid (FAI) produced in house from adult Swiss albino mice (*Mus musculus*) by SAARB [36] and incubated in a humid chamber and oven (NAPCO) at 37 °C and 5% CO_2_ for 30 min. After incubation, the slides were immersed in 1X PBS (phosphate-buffered saline) solution (pH 7.2) for 10 min at 25 °C and then rinsed in distilled water. Then, 25 μL of fluorescein isothiocyanate-conjugated anti-mouse antibody (fluorescein-conjugated goat IgG fraction to mouse immunoglobulin: IgG, IgA, IgM; Cappel, United States) diluted 1:900 in PBS, also containing 0.5% Evans Blue dye, was added to each well of the slide. Slides were incubated for an additional 30 min in a humidity chamber and oven, followed by immersion in PBS and subsequent rinsing with distilled water. To fix the coverslip, 25 μL of buffered glycerol (pH 8.2) was added. Finally, they were observed using a fluorescence microscope (Olympus BX51) with a UPlanFL N20X/0.5 objective and WB and U-25nd filters [37].

The degree of fluorescence of the positive specimens was graded from 1+ (one cross) to 4+ (four crosses), where 1+ is minimal fluorescence and 4+ is intense fluorescence [38].

### 2.10. Infection, Dissemination, and Transmission Analysis

Infection, dissemination, and transmission rates were calculated for three dpi measurements. The infection rate was determined by dividing the number of females with an infected body by the total number of females analyzed on the corresponding dpi. The dissemination rate was based on the number of females with an infected head among those with an infected body on the analyzed dpi. And the rate of transmission was calculated by counting the number of females with positive saliva tests who also tested positive for both body and head infections.

## 3. Results

Two infections were performed using F1 females from the same colony, with a total of 70 females analyzed. Of those, 16 females were from the first infection and 54 females were from the second infection.

On the 7th dpi, 4 females from the first infection and 17 females from the second infection were segmented. On the 14th dpi, another 4 females were segmented from the first infection and 18 females were segmented from the second infection. On the 21st dpi, a total of 8 females were segmented from the first infection and 19 females were segmented from the second infection.

Females from each dpi were analyzed individually and three samples (body, head, and saliva) were collected. First, an attempt was made to isolate the body and head samples, and if both samples were positive, indicating the presence of infection and dissemination of the virus, the saliva was analyzed to determine the likelihood of transmission.

**Figure 1:**
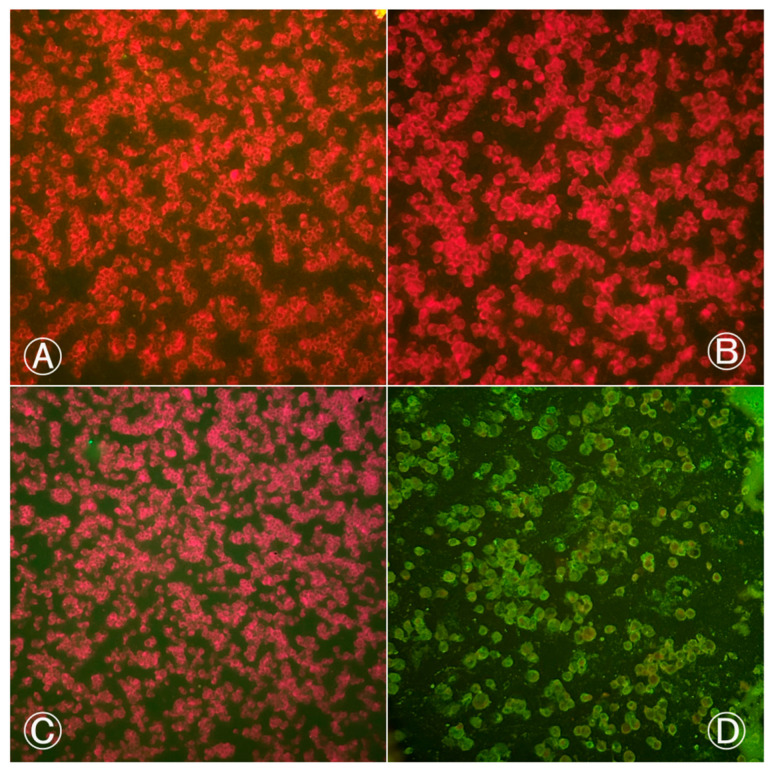
Indirect immunofluorescence of *Culex quinquefasciatus* (Ananindeua/PA) body and head samples exposed to blood meal infected with BeH 7445 strain (ILHV): (**A**) head sample from the 2nd infection procedure, female; (**B**) body sample from the 1st infection procedure, female; (**C**) body sample from the 2nd infection procedure, female; (**D**) positive control with ILHV-infected cells. Cells marked in red by Evans Blue dye indicate negative cells. Cells marked in fluorescent green by fluorescein indicate positive cells. Images were taken at 200× magnification.

Based on the results of the IFI test, *Cx*. *quinquefasciatus* proved to be resistant to the BeH 7445 strain of ILHV, as none of the body and head samples were positive for virus at any of the dpi’s evaluated (Figure 1).

**Figure 2:**
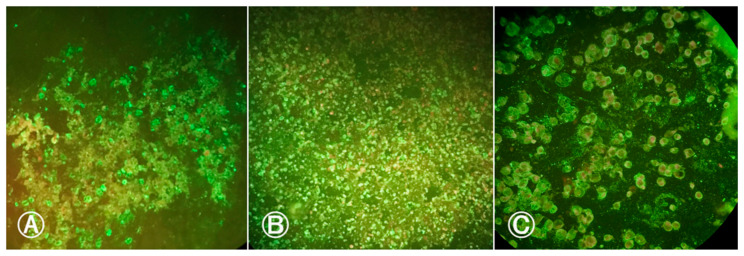
Indirect immunofluorescence of infected blood meal samples with BeH 7445 strain (ILHV) offered to females: (**A**) Blood meal sample from first infection procedure showing ILHV positive cells; (**B**) blood meal sample from the second infection procedure showing positive cells for ILHV; (**C**) positive control with ILHV-infected cells. Cells marked in red by Evans Blue dye indicate negative cells. Cells marked in fluorescent green by fluorescein indicate positive cells. Images were taken at 200× magnification.

Given the negativity of the head and body samples, we attempted to verify the virus presence in the blood that was offered to the females during oral infection. We accomplished this by conducting the IFI test on an aliquot of the infected blood from both infections, which yielded positive results for both blood samples (Figure 2). The infected blood from the first infection procedure exhibited two crosses (++) of fluorescence, while the infected blood from the second infection displayed four crosses (++++).

We analyzed the titers of infected blood samples from both infections. These showed titers of 1.45 × 10^6^ PFU/mL for the first infection and 1.55 × 10^6^ PFU/mL for the second infection.

As a result, the ILHV infection and dissemination rates were 0% in both infections and in all three dpis analyzed. Therefore, due to the negativity of the body and head samples, indicating no spread of ILHV, we did not analyze the saliva samples.

## 4. Discussion

The population of *Cx*. *quinquefasciatus* (Ananindeua/PA) demonstrated refractoriness to ILHV, as none of the body and head samples yielded positive results in the IFI test, even after exposure to higher viral titers.

We would also like to point out that this is the first time that the population of *Cx*. *quinquefasciatus* (Ananindeua/PA) has been analyzed for vector competence for ILHV and that in a previous study [39], it had already been shown to be a competent vector for the transmission of *Orthoflavivirus nilense* (WNV), with detection of the virus using the same diagnostic test in body, head, and saliva samples, indicating that this population is capable of becoming infected with WNV, allowing it to spread and also transmit the virus.

However, this is not the first record of resistance of Brazilian populations of the species *Cx*. *quinquefasciatus* to some arboviruses in which infected *Culex* mosquitoes collected in the field have already been detected, indicating that the isolation of arboviruses in field samples does not necessarily indicate the participation of this vector in the transmission cycle, since several factors must be taken into account, such as the presence of swollen females in the pools analyzed with the possibility of a recent blood meal in a viremic host.

In a study conducted by Fernandes [40], populations from the cities of Recife (PE), Campina Grande (PB), and Rio de Janeiro (RJ) were also refractory to different strains of ZIKV, with an infection rate of 0% for the ZIKVPE243 and ZIKVSPH strains and only 5% for the ZIKVU1 strain, and in relation to the dissemination rate, it was 0% for the three strains analyzed in the study.

Lourenço-de-Oliveira et al. [41] evaluated the ability of two Brazilian strains of *Cx. quinquefasciatus* to be infected with ZIKV via blood meal, with one strain previously infected with *Wolbachia pipientis* (wPip) and the other not. In their experiments, the group found that both strains were resistant to ZIKV, regardless of the presence or absence of the wPip bacterium, and the virus was not detected in body, head, and saliva samples evaluated at 7 and 14 days.

Regarding *Mayaro virus* (MAYV), the *Cx*. *quinquefasciatus* species from Belo Horizonte (MG) city which was orally infected with the TRVL 4675 strain of MAYV, demonstrated resistance to the virus even when the concentration of the virus was raised. At a viral titer of 10^9^ PFU/mL, the infection rate was 0%. When the titer was increased to 6 × 10^9^ PFU/mL, there was a minimal increase to 5%. Only one female out of twenty tested positive for the head and thorax mix, while none of the saliva samples from the species were positive for MAYV [42].

The vector competence of the species for *Orthoflavivirus denguei* (DENV) was also evaluated in 1992 [43], in which *Cx*. *quinquefasciatus* mosquitoes from the city of San Juan (Puerto Rico) were parentally infected with DENV and evaluated individually on the 2nd, 4th, 7th, 9th, 11th, and 15th dpi, also showing absence of the virus in the head samples of the mosquitoes of the species.

The vector competence of *Cx. quinquefasciatus* for *Orthobunyavirus oropoucheense* (OROV) was evaluated using mosquito populations from Belo Horizonte. Mosquitoes were orally infected with the prototype strain BeAn19991 and OROV RNA was not detectable in all samples collected at 7th and 14th days post infection, indicating the refractoriness of the species to OROV [44].

The susceptibility and refractoriness of mosquitoes to a pathogen involve various processes that impact vector competence. These processes include the innate immune response, gene expression, and virus dissemination. Some mosquito strains prove refractory to pathogens when they migrate to regions that do not allow replication due to “physiological incompatibility”, whereby viral antigens cannot bind to cell receptors in that region [45,46].

However, it is just as important to investigate how genetic flexibility of pathogenic agents affects vector capacity because genetic inheritance of pathogenic agents may be closely related to vector response to infection [45,46].

## 5. Conclusions

Based on the results of the study, it appears that *Cx*. *quinquefasciatus* (Ananindeua/PA) is resistant to *Orthoflavivirus ilheusense*, regardless of the virus titer to which it was orally exposed. Thus, the lack of virus detection in the body and head samples at all three evaluated dpis (7th, 14th, and 21st dpi) suggests that this species may not act as a vector in the *Orthoflavivirus ilheusense* (ILHV) transmission cycle. This study represents the first evaluation of the vector competence of *Culex* mosquitoes for the transmission of ILHV arbovirus in light of the various reports of virus isolation in *Culex* samples collected in the field. Thus, we emphasize the need for further research to determine whether *Cx*. *quinquefasciatus* is really a vector for this arbovirus in order to understand other aspects that influence the vector’s competence, such as comparison with other strains of ILHV from different hosts, as well as the analysis of other mosquito strains, as well as the molecular and genetic study of pathogen–vector interaction, such as interaction with mosquito cell receptors, the action of infection barriers, and the genetic and immune response to infection.

## Data Availability

The data presented in this study are available in the article.
